# Loss of nuclear envelope bud formation leads to mitophagy initiation in *Drosophila* muscles

**DOI:** 10.1080/27694127.2025.2471121

**Published:** 2025-03-04

**Authors:** Yungui Guo, David Brooks, Ziwei Zhao, Erica Biven, Erika R. Geisbrecht

**Affiliations:** Department of Biochemistry and Molecular Biophysics, Kansas State University, Manhattan, KS, USA

**Keywords:** Muscle, autophagosome, mitochondria, Nuclear envelope budding, *D. melanogaster*

## Abstract

Pavarotti (Pav) and its binding partner Tumbleweed (Tum) are well known for their evolutionarily conserved roles in microtubule-dependent movements during cytokinesis. In post-mitotic *pav RNAi* muscles, we unexpectedly observed the accumulation of puncta marked by ubiquitin, p62, and Atg8a without an obvious disorganization of the microtubule network. Some of these autophagosomal structures clustered together and colocalized with mitochondria. The Pav-Tum complex was enriched in muscle nuclei, consistent with roles for Pav and Tum in nuclear envelope (NE) budding, an alternative pathway for the export of large ribonucleoproteins. One of the established cargoes of the *Drosophila* NE budding pathway, *Marf mRNA*, was indeed reduced in the myoplasm of *pav RNAi* muscles. Moreover, RNAi knockdown of Marf or the NE budding components Wash or Torsin also caused the clustering of p62-marked mitochondria. These data together define a model whereby blocking NE budding reduces mitochondrial activity and in turn recruits p62 and autophagic structures for a lysosomal fate.

## Introduction

Macroautophagy (hereafter referred to as autophagy) is generally considered a non-selective, bulk degradation system whereby portions of the cytoplasm and organelles are delivered to the lysosome for degradation and recycling [1,2]. In contrast, damaged or harmful cargo are eliminated through a selective autophagy process that is dependent on ubiquitin-dependent autophagy receptors [3]. Both forms of autophagy require phagophore formation, expansion, and closure, resulting in double-membraned autophagosomes that undergo eventual fusion with the lysosome.

GTPases of the dynamin family mediate dynamic mitochondrial fission and fusion, with fission dividing mitochondria into two and fusion uniting mitochondria. Mitochondrial assembly regulatory factor (Marf) is responsible for mediating outer mitochondrial fusion, while Dynamin-related protein 1 (Drp1) catalyzes the constriction of this organelle towards fission [4]. These dynamics are crucial for the regulation of mitochondrial quality. Fragmented or nonfunctional organelles are continually removed by basal mitophagy, while other factors, including aging, oxidative stress, or mutations, promote mitochondrial damage and trigger increased mitochondrial turnover [5,6]. Mitophagy is one form of selective autophagy that prevents the accumulation of damaged mitochondria [7]. The ability of mitochondria to undergo dynamic fission and fusion further highlights the need for a selective removal process as the fusion of impaired mitochondria with their healthy counterparts can drastically affect mitochondrial, and therefore cellular, energy outputs [8].

Proper transport and local translation of nuclear-encoded mitochondrial transcripts is one factor that affects mitochondrial dynamics and health [9]. In addition to the export of mRNAs through nuclear pore complexes (NPCs), some mitochondrial transcripts are transported through nuclear envelope (NE) budding, a recently described alternative pathway for cargo that are too big to traverse through nuclear pores [10,11]. Originally characterized as a route for herpesvirus export from the nucleus [12], it is now clear that NE budding is an endogenous mechanism of nuclear transport across multiple species, including *Saccharomyces cerevisiae, Drosophila melanogaster*, and *Homo sapiens*. Studies in *Drosophila* have largely focused on the requirement for NE budding in the transport of large ribonucleoprotein (megaRNP) complexes during larval neuromuscular junction (NMJ) development and for mitochondrial integrity in the adult indirect flight muscles (IFMs) [13–15]. For example, *par6* transcripts are found at foci corresponding to NE buds as well as the postsynaptic larval NMJ [13]. Additionally, mRNAs for *Marf* and other mitochondria-associated transcripts are present in NE bud foci in larval muscles [14].

Pavarotti (Pav)/kinesin family member 23 (Kif23) and its binding partner Tumbleweed (Tum)/RacGAP1 comprise the centralspindlin complex, which organizes microtubule (MT) arrays at the mitotic spindle during cytokinesis [15]. Recently, these two proteins were also shown to be enriched in NE bud foci and required for the nuclear budding process through physical interactions with Wiskott-Aldrich Syndrome protein (WASH) [15]. Here we present new data that RNA interference (RNAi) knockdown of Pav or Tum in larval muscles results in the aggregation of p62-marked structures that colocalize with mitochondria. *pav RNAi* muscle nuclei show fewer NE buds and reduced *Marf mRNA* in the myoplasm. Moreover, the aggregation of mitochondria and overlap with p62 are phenocopied in *Marf RNAi* muscles. We propose that loss of *Marf*, and possibly other transcripts, reduce mitochondrial integrity which in turn recruits ubiquitin and p62 for autophagosome formation.

## Results

### Loss of Pav results in elevated p62 puncta in larval bodywall muscles

**Figure 1.**
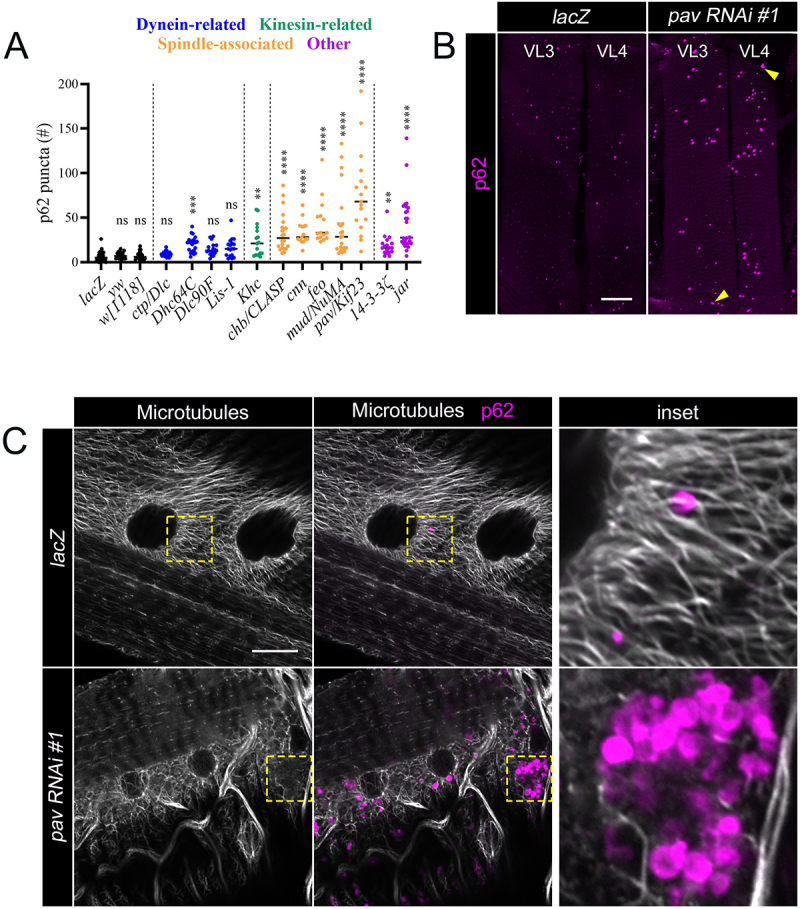
Targeted genetic miniscreen reveals increased p62 puncta in *pav RNAi* muscles. (A) Scatter plot quantifying the number of p62(+) puncta for *Mef2*-Gal4 driven expression of UAS*-lacZ* control or UAS*-candidate RNAi* lines. *yw* and *w[1118]* were included as genetic background controls for RNAi knockdown constructs. Each data point represents the total number of p62 puncta in VL3 and VL4 muscles of L3 larvae. P-values: ns, not significant; **, p<0.01; ***, p<0.005; ****, p<0.001. The median is shown as a solid black line. N ≥15. (B) Maximum intensity projections of VL3 and VL4 muscles immunostained for p62 in *lacZ* control or *pav RNAi #1* muscles from L3 larvae reveal an increase in the number of p62 puncta (magenta). p62 clusters are indicated by the yellow triangle. (C) High magnification single plane images of control or *pav RNAi #1* VL3 muscles co-stained with anti-p62 (magenta) and anti-α-tubulin (gray). Inset depicts the area of the yellow dashed box. Scale bars, 40 µm (panel B), 15 µm (panel C).

To discover new autophagy regulators, we performed a mini RNAi screen that targeted proteins with microtubule or trafficking-related functions using *Drosophila* larval muscles as a cell biological model. UAS-RNAi lines were crossed with the *Mef2-Gal4* driver to selectively knock down target transcripts in muscle tissue. The selected genes were classified into four groups based upon predicted functions of the targeted proteins - dynein-related, kinesin-related, spindle-associated, and other (Figure 1A). Wandering third instar larva (L3) were filleted and the resulting muscle carcasses were immunostained with an antibody generated against Ref(2)p/p62. p62 is a receptor for ubiquitinated cargo that could potentially be captured by autophagosomes and degraded by autophagy [16,17]. Quantitation was performed using the Analyze Particles function in ImageJ and the results reported reflect the total number of p62(+) puncta in the ventral longitudinal 3 (VL3) and ventral longitudinal 4 (VL4) muscles. In comparison to the negative control *Mef2>lacZ*, we observed a significant increase (p<0.01) in the number of p62(+) puncta in 9 out of the 12 RNAi lines tested. We chose to focus on Pav/Kif23 as RNAi knockdown resulted in the strongest phenotype compared to other RNAi lines, with approximately 3.6 fold more p62(+) puncta than control muscles (Figure 1B and Figure S1B). We were also intrigued by the presence of p62(+) clusters (yellow triangles) in these *pav RNAi* muscles (Figure 1B). While we have analyzed other muscle mutants with elevated p62 puncta, this novel clustering phenotype has not been observed [18,19].

Since Pav belongs to the kinesin superfamily and is a MT motor protein [20], we next immunostained for MTs in *pav RNAi* muscles. The overall MT pattern was similar in *pav RNAi* and *lacZ* muscles (Figure 1C and Figure S1C), indicating that a reduction in Pav function does not grossly alter MT organization. From high magnification images where the MTs and p62 were co-labeled, we observed p62(+) puncta associated with MTs in *lacZ* muscles (Figure 1C). However, in *pav RNAi* muscles, the MT staining was fainter and appeared to be excluded from the aggregated p62 structures. We analyzed a second, independent *pav RNAi* (*pav RNAi #2*) line and indeed, the number of p62(+) puncta in VL3/VL4 muscles was increased ~2 fold compared to *lacZ* controls (Figure S1A,B) and also contained p62 clusters (yellow triangles).

## Autophagosomal structures accumulate in *pav RNAi* and *tum RNAi* muscles

*Drosophila* Tum is a binding partner of Pav during cytokinesis and NE budding [15,21], but Pav and Tum also have independent roles in embryo wound repair and oogenesis [22]. Therefore, we tested whether Tum is also required to prevent the aggregation of p62(+) structures. Immunostaining of p62 in two, independent *tum RNAi* lines replicated both the increase and clustering of p62 puncta (yellow triangles) seen in *pav RNAi* muscles (Figure S1A). The total number of p62(+) puncta was increased ~2.6-fold in *tum RNAi #1* and ~4.6-fold for *tum RNAi #2* compared to *lacZ* control muscles (Figure S1B). Verification of RNAi knockdown in our original *pav* and *tum RNAi* lines was performed using qPCR. Indeed, *pav mRNA* levels were reduced ~70% in the *pav RNAi* line compared to the control (Figure S1D) and *tum* transcripts were reduced ~60% in the *tum RNAi* line (Figure S1E). This data confirm the functionality of the *pav* and *tum RNAi* lines and demonstrate that both components of the centralspindlin complex have similar roles in preventing the formation of p62 clusters.

**Figure 2.**
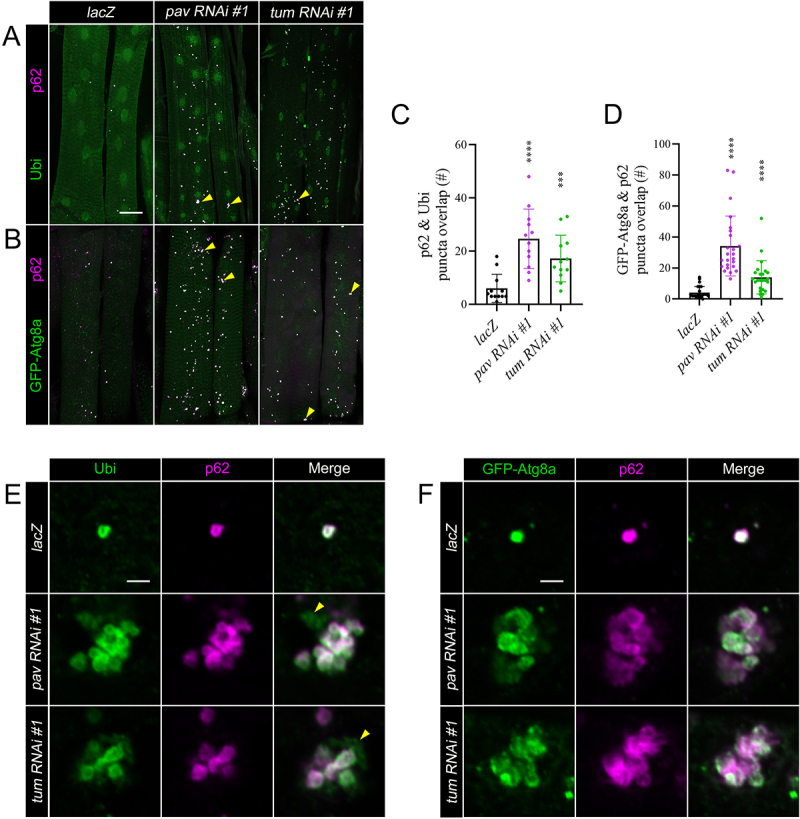
p62-Ubi and Atg8a-p62 complexes accumulate in *pav* and *tum RNAi* muscles. (A and B) Maximum intensity projections of VL3 and VL4 muscles in *lacZ* control, *pav RNAi*, or *tum RNAi* muscles driven by *Mef2-*Gal4. (A) Following Pav or Tum knockdown, there is an appreciable increase in Ubi (green) and p62 (magenta). p62(+) and Ubi(+) clusters are present in *pav* and *tum RNAi* muscles (yellow triangles). (B) There are more clusters marked by p62-Atg8a in *pav RNAi* or *tum RNAi* muscles also expressing GFP-Atg8a (green) and co-labeled with anti-p62 (magenta) compared to *lacZ* controls. Clusters marked by p62 and GFP-Atg8a are denoted by yellow triangles. (C and D) Scatter bar graphs depict an increase in the number of puncta in VL3 and VL4 muscles that show overlap between p62 and Ubi (C) or GFP-Atg8a and p62 (D) in *pav* or *tum RNAi* muscles compared with controls. Each data point represents the total number of puncta that exhibit p62-Ubi or Atg8a-p62 in VL3 and VL4 muscles of L3 larvae. P-values: ***, p<0.005; ****, p<0.001. Error bars indicate standard deviation (SD). N=12 for C and N=24 for D. (E and F) Single plane, high magnification images of p62(+) clusters in *pav* and *tum RNAi* L3 muscles. (E) Individual p62-Ubi puncta in *lacZ* muscles or a representative p62-Ubi cluster in *pav* and *tum RNAi* muscles. The yellow triangle shows regions where Ubi (green) is not recognized by p62 (magenta). (F) In *lacZ* muscles, p62 staining overlaps with GFP-Atg8a signal (*Mef2>GFP-Atg8a*) in a single mitochondrion, whereas the overlap appears as clusters in *pav* and *tum RNAi* muscles. Scale bars, 40 µm (panel A), 2 µm (panels E and F).

Ubiquitin (Ubi) chains appended to proteins and/or organelles confer a degradation signal directed to the proteasome (typically K48-linked chains) or the lysosome (typically K63-linked chains) [23]. In the latter case, the Ubi receptor p62 recognizes K63-linked Ubi chains and directly recruits Autophagy-related protein 8 (Atg8a)/Microtubule-associated protein 1A/1B-light chain 3 (LC3) to form the autophagosome [24]. If p62 serves as an autophagy receptor, it would be expected to colocalize with Ubi and Atg8a on forming autophagic membranes (phagophores) or label mature autophagosomes. Indeed, the number of structures that stained positive for both Ubi and p62 was increased in *pav* and *tum RNAi* muscles compared with controls (Figure 2A). The total number of puncta marked by p62 and Ubi was increased ~4-fold in *pav RNAi* muscles and ~2.8-fold in *tum RNAi* muscles over that of *lacZ* (Figure 2C). These data are consistent with the fold increases seen in p62 immunostaining alone (Figure S1B). High magnification images in control or *pav* and *tum RNAi* muscles showed a near perfect overlap of Ubi and p62 staining (Figure 2E). Occasionally in muscles with *pav* or *tum RNAi* knockdown, structures with Ubi signal that lack p62 staining were observed (yellow triangles). These regions likely represent Ubi-tagged proteins and/or organelles that have not yet recruited p62. Note areas lacking p62 are found on the outside edges of Ubi clusters, suggesting that p62 may be key for initiating or promoting aggregation.

To verify whether the Ubi-p62 complexes recruit Atg8a, we next assessed p62 and Atg8a colocalization in *pav RNAi* and *tum RNAi* muscles. We were unable to perform double labeling using anti-p62 and anti-Atg8a because both antibodies were generated in the same species. Therefore, we took advantage of a green fluorescent protein (GFP)-tagged Atg8a fusion protein (GFP-Atg8a) and used an anti-GFP antibody to enhance Atg8a signal. Autophagosomal structures marked by Atg8a were indeed positive for p62 signal in *pav RNAi* or *tum RNAi* muscles compared with controls (Figure 2B). The average number of puncta with both Atg8a and p62 signal was ~8-fold or ~4-fold in *pav RNAi* or *tum RNAi* muscles, respectively (Figure 2D). Additional analysis showed consistent overlap of GFP-Atg8a and p62 in clusters (yellow triangle) upon knockdown of Pav or Tum (Figure 2B,F). These data together show that Ubi, p62, and Atg8a are consistently recruited to punctate clusters in muscles upon a decrease in Pav or Tum function.

The quantity of autophagosomes detected at any specific time reflects a balance between their generation and their degradation in lysosomes. To investigate if the increase in autophagosomal structures marked by Atg8a in *pav RNAi* or *tum RNAi* muscles are due to altered autophagosome and/or lysosome biogenesis, we examined transcript levels of key genes required for the production of these organelles [25,26] in control (*y,w* and *mCherry RNAi*) or *pav RNAi* larvae. The mammalian transcription factor EB (TFEB), or melanocyte-inducing transcription factor (Mitf) in *Drosophila*, is a master gene for autophagosomal and lysosomal biogenesis [27]. However, *Mitf mRNA*, as well as its transcriptional targets *Autophagy-related 1 (Atg1), Autophagy-related 18 (Atg18a)*, and *Cysteine proteinase-1 (Cp1)*, were not significantly altered in *pav RNAi* muscles (Figure S2A). This data together show that knockdown of Pav does not likely enhance autophagosome (Atg1, Atg18) or lysosomal biogenesis (Cp1) via a transcriptional mechanism.

**Figure 3.**
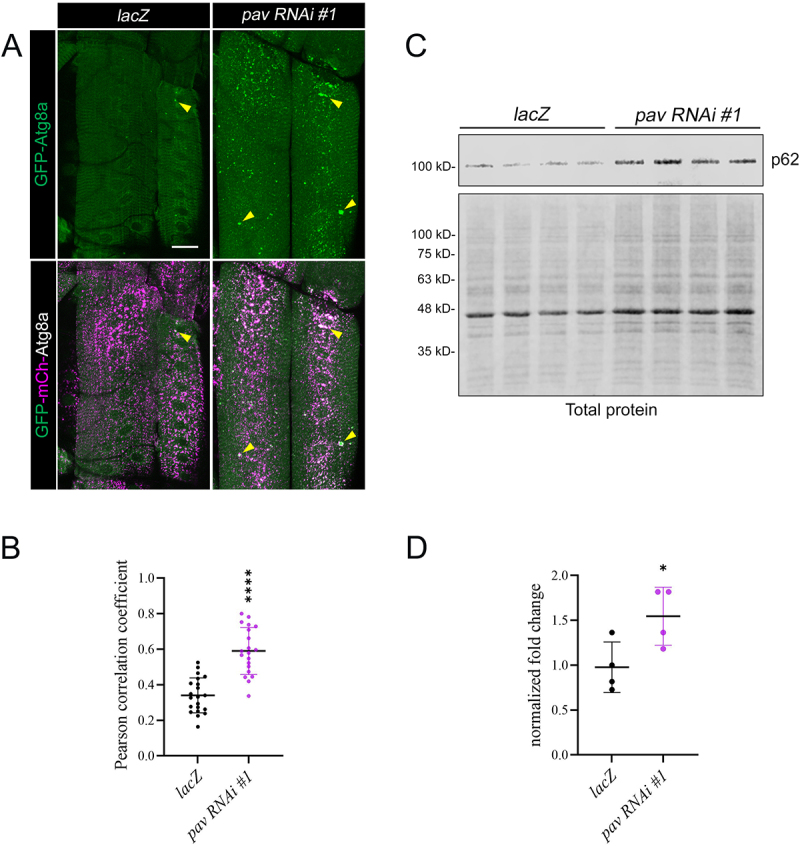
Stalled autophagic structures accumulate in Pav knockdown muscles. (A) Maximum intensity projections showing expression of the tandem GFP-mCh-Atg8a fusion protein in VL3 and VL4 muscles (*Mef2>GFP-mCh-Atg8a*). Increased colocalization of GFP and mCh fluorescence (white puncta denoted by yellow triangles) indicates that the tandem protein localizes to an autophagic structure that have not fused with lysosomes. (B) Scatter plot quantifying the increased overlap between GFP and mCh fluorescence measured by the Pearson correlation coefficient (PCC). Values closer to 1.0 indicate more co-localization. Each data point represents PCC values in different muscles. Two images were taken from ten different VL3 or VL4 muscles. P-value: ****, p<0.001. Error bars indicate standard deviation (SD). N≥20. (C) Western blot showing p62 levels in dissected muscle carcasses for *lacZ* control or *pav RNAi* muscles. Total protein staining is used as a loading control. N=4 biological replicates. (D) Quantitation of the Western blot data (from panel C) shows increased levels of total p62 protein in muscle carcasses after Pav knockdown. Each data point represents an independent biological replicate. P-value: *, p<0.005. Error bars indicate standard deviation (SD). N=4. Scale bar, 40 µm (panel A).

An alternative explanation for the accumulation of Ubi(+)/p62(+)/Atg8a(+) puncta in muscles lacking Pav or Tum is a reduction in autophagosomal turnover, possibly due to abnormal lysosomal activity or the impairment of transport and/or fusion with the lysosome. We first tested if lysosome number or activity were altered by measuring the fluorescence of the lysosomal protease Cathepsin B. These experiments showed no difference between control or *pav RNAi* muscles (Figure S2B,C). To measure autophagic activity, we expressed a tandem fluorescently-tagged Atg8a, GFP-mCherry (mCh)-Atg8a, in *lacZ* or *pav RNAi* control muscles. The acid-labile GFP fluorescence is quenched in the acidic lysosomal environment and labels forming isolation membranes (phagophores) or mature autophagosomes, while mCh fluorescence marks autolysosomes or lysosomes. *Pav RNAi* muscles expressing GFP-mCh-Atg8a showed increased green (GFP) and white (GFP/mCh) puncta, including clusters (yellow triangles), compared to *lacZ* control muscles (Figure 3A). Analysis of colocalization using the Pearson correlation coefficient (PCC) confirmed more overlap between the GFP and mCh signal in *pav RNAi* muscles (Figure 3B and Figure S2D,E), pointing to an increase in autophagosomal structures that failed to undergo lysosomal fusion and turnover.

As a second, independent method to probe autophagy, we assessed the levels of p62 in muscle fillets. Since p62 is bound to ubiquitinated proteins and becomes incorporated into autophagosomes which traverse to the lysosome for degradation, p62 protein levels inversely correlate with pathway activity [28]. Immunoblotting (Figure 3C) of four independent biological replicates showed elevated levels of total p62 protein in *pav RNAi* samples compared to controls (Figure 3D). Note there was no change in *p62* transcript levels in *da>pav RNAi* larva (Figure S2A), thus ruling out increased gene expression as an underlying cause for p62 accumulation. These data are consistent with our GFP-mCh-Atg8a reporter and together indicate a block in the turnover of Atg8a(+) immature or mature autophagosomes.

## Pav and Tum function in the nucleus

Since autophagosomal structures accumulate in *pav* or *tum*-deficient muscles, we were curious about the subcellular distribution of Pav and Tum in the myoplasm. Despite trying different immunostaining methods, we failed to detect signal in muscle tissue using available anti-Pav and anti-Tum antibodies [15]. Therefore, we took advantage of GFP-tagged fusion proteins for Pav (Ubi-GFP-Pav) or Tum (sqh-Tum-GFP) and enhanced the signal using an anti-GFP antibody. While no signal was observed in the myoplasm, strong signal for GFP-Pav (Figure S3A) and GFP-Tum (Figure S3B) were observed in the nuclei of numerous larval tissues, including muscle, fat body, gut, salivary gland, and the wing disc. This finding is consistent with previous reports of Pav or Tum localization in the *Drosophila* epidermis and ovary [29,30] and point towards a role for the centralspindlin complex originating in the nucleus.

**Figure 4.**
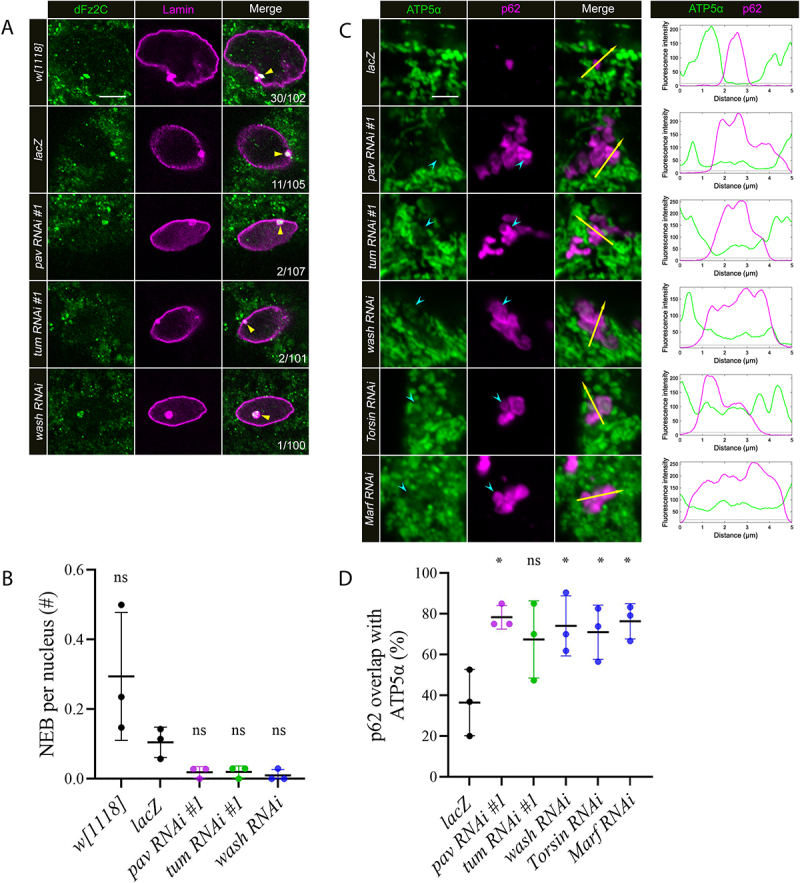
Knockdown of Pav or Tum decreases the formation of NE buds resulting in p62 clusters that overlap with ATP5α. (A) Progeny from *Mef2-*Gal4 flies mated with UAS*-lacZ*, UAS*-pav RNAi #1*, UAS*-tum RNAi #1*, or UAS*-wash RNAi* are analyzed here. *w[1118]* is an additional control. Single plane images of representative nuclei from VL3 or VL4 muscles of the specified genotypes were stained with anti-Lamin and anti-dFz2C antibodies. NE buds correspond to foci that stain positive for both Lamin (magenta) and dFz2C (green) and are indicated by the yellow triangles. The number of buds that stain positive for Lamin/dFz2C out of the total number of nuclei counted are shown in each panel. (B) Scatter plot quantifying the number of NE buds per nucleus. Each dot represents one group containing N≥33 nuclei. P-value: ns, not significant. Error bars indicate standard deviation (SD). N=3 groups for each genotype. (C) Confocal projections of muscles co-stained with anti-p62 (magenta) and anti-ATP5α (green) in the indicated genotypes. An example of mitochondrial signal that overlaps with p62 is denoted by the cyan indented arrowhead. The corresponding fluorescence plots were produced using the ImageJ Plot Profile function which quantifies the fluorescence intensity across the trajectory of the yellow arrow. (D) Scatter plot quantifying the percentage of p62(+) puncta that overlaps with anti-ATP5α staining in the indicated genotypes. Each data point corresponds to a single group of at least N≥19 p62(+) puncta. P-values: ns, not significant; *, p<0.005. Error bars indicate standard deviation (SD). N=3 groups for each genotype. Scale bars, 6 µm (panel A), 3 µm (panel C).

A recent study described new functions for Pav and Tum in the formation of NE buds [15]. To visualize these structures in larval muscle nuclei, we immunostained using the only two markers available, the C-terminal fragment of the *Drosophila* Wingless receptor dFz2 (dFz2C) or Lamins [13–15,31,32]. Thus, colocalization with both anti-dFz2C and anti-Lamin B were used as criteria for counting the number of NE foci/nucleus (Figure 4A, yellow triangle). We identified 30 NE buds distributed among 102 nuclei (0.29 foci/nucleus) in *w^1118^* muscles and 11 NE buds were present in 105 nuclei (0.10 foci/nucleus) in *lacZ* muscles (Figure 4B). In contrast, only 1-2 instances of NE bud visualization were observed in muscle nuclei after RNAi knockdown for Pav (0.01 foci/nucleus), Tum (0.01 foci/nucleus), or Wash (0.01 foci/nucleus) (Figure 4A). The persistence of any NE buds is likely attributed to incomplete knockdown using the RNAi approach as we know there is remaining *pav* and *tum* transcripts via RT-PCR (Figure S1D,E).

## Nuclear roles for Pav and Tum influence mitochondrial and autophagosomal clustering

Autophagosomes can engulf varied intracellular constituents, including pathogens, impaired proteins, and organelles, before being conveyed to lysosomes for degradation [1,7]. To determine the type of cargo inside the autophagosomal clusters in *pav* and *tum RNAi* muscles, we performed co-labeling experiments for p62 and candidate protein targets using available antibodies in our laboratory. Eventually we found that the aggregated p62 staining overlapped with weak signal corresponding to ATP-Synthetase α (ATP5α), (also known as ATP synthase F1 subunit alpha, or ATP5A) [33].

We used ImageJ to quantitate the intensity of ATP5α (green) and p62 (magenta) signal as a measure of overlap (yellow arrows in Figure 4C) in multiple RNAi genotypes that block the formation of NE buds. Most p62 signal did not overlap with the ATP5α immunostaining in *lacZ* muscles, but provided intensity levels for background (~5, dotted gray line) and maximal (~200) fluorescence intensities. Using this line plot method, we confirmed that ATP5α overlapped with p62 clusters (cyan arrowheads) in *pav RNAi, tum RNAi* and *wash RNAi* muscles (fluorescent intensity ~50). Approximately 36% of p62 signal colocalized with ATP5α staining in *lacZ* muscles, but this percentage of overlap increased to ~78% in *pav RNAi*, ~67% in *tum RNAi*, and ~74% in *wash RNAi* muscles (Figure 4D). Previous research has shown that TorsinA is involved in the formation of NE buds in mouse neuronal nuclei [34]. Since *Drosophila* Torsin also mediates the transport of megaRNPs via NE budding [31], we examined the consequences of *Torsin RNAi* in larval muscles. Similar to the RNAi phenotypes in Pav, Tum, or Wash muscles, we found that p62 overlapped with ~71% of the ATP5α(+) clusters upon the induction of *Torsin RNAi* (Figure 4C, D).

In all NE budding genotypes that exhibited p62 overlap with weakly-stained, clustered mitochondria, the majority of surrounding organelles retained brighter ATP5α staining (Figure S4A). We hypothesized that this weaker ATP5α signal may indicate a reduction in mitochondrial mass and/or activity [15]. To further probe this idea, we first treated *lacZ* control and *pav RNAi* muscles with tetramethylrhodamine methyl ester (TMRM) and MitoTracker (Figure S4B). The accumulation of TMRM in mitochondria with intact membrane potentials yields strong fluorescence. When mitochondrial membrane potential drops, TMRM signal declines or disappears. In randomly selected regions of muscles, we used ImageJ to measure the fluorescence intensities of MitoTracker and TMRM signal to calculate an intensity ratio. Since the majority of mitochondria stain brightly for ATP5α or MitoTracker (Figure S4A, B), quantitation revealed no overall difference in the TMRM/MitoTracker ratio between *pav RNAi* or *lacZ* muscles (Figure S4C). However, TMRM signal was absent in areas where the MitoTracker staining was also weak in *pav RNAi* muscles (inset in Figure S4B). Expression of DsRed with a mitochondrial targeting sequence showed a similar result. Clusters of p62 (cyan arrowheads) overlapped with both anti-ATP5α and DsRed-Mito stainings (Figure S4D). These data together suggest that mitochondrial mass and activity are lower in clusters marked by p62(+) in genotypes that block NE bud formation.

The mitochondrial morphology of p62(+)/ATP5α(+) clusters in RNAi muscles that restrict NE bud development resembles the smaller, spherical mitochondria upon RNAi knockdown of *Drosophila* Marf in either larval or adult IFMs [35-37]. Therefore, we performed anti-p62 and anti-ATP5α colabeling in *Marf RNAi* muscles and indeed, observed the same unique clustered p62(+)/ATP5α(+) phenotype evident in *pav, tum, wash*, or *Torsin RNAi* muscles (Figure 4C). The ratio of p62 signal that overlapped with anti-ATP5α staining was also elevated in *Marf RNAi* muscles (Figure 4D). This result is consistent with muscles defective in NE budding and suggests that normal Marf function may be important to prevent mitochondrial clustering and p62 accumulation.

**Figure 5.**
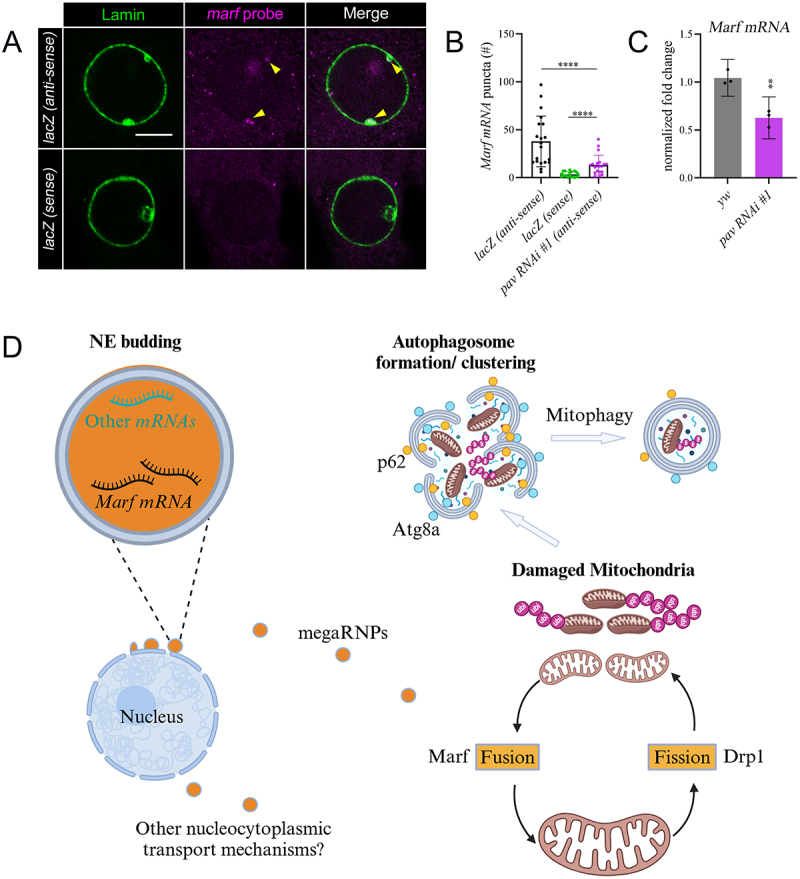
FISH and qPCR experiments demonstrate a reduction in *Marf mRNA* export and transcript levels in *pav RNAi* muscles. (A) Single plane confocal images of *lacZ* muscles incubated with the *Marf* anti-sense probe and stained with Lamin shows colocalization of *Marf mRNA* (magenta) with budding foci at the NE (green) indicated by the yellow triangle. (B) Scatter bar graph shows that the number of foci containing *Marf mRNA* in *lacZ* control muscles is greater than in *pav RNAi* muscles. Each data point represents the number of puncta labeling *Marf* transcripts in VL3 and VL4 muscles. P-value: ****, p<0.001. Error bars indicate standard deviation (SD). N=20. (C) qPCR shows that *Marf* transcript levels are significantly reduced in *pav RNAi* compared with controls. P-value: **, p<0.01. Error bars indicate standard deviation (SD). N= 3 biological replicates. (D) A proposed model for *Marf mRNA* export from NE buds to regulate mitochondrial morphology and health. Made in Biorender.com. Scale bars, 6µm.

*Marf mRNA* is a known cargo of NE buds [14]. Since suppression of NE buds in *pav* or *tum* RNAi shows a mitochondrial clustering phenotype consistent with the knockdown of *Marf* expression, we postulated that the export of *Marf* transcript from the nucleus to the myoplasm may be compromised upon a reduction in Pav function. Therefore, we conducted fluorescence in situ hybridization (FISH) to validate our hypothesis. FISH employs fluorescent probes to precisely determine the intracellular location of labeled nucleic acids within a cell. We generated an anti-sense probe to detect *Marf mRNA* and a sense probe to serve as a non-specific control. As expected, we detected *Marf* transcript in NE buds. In *lacZ* muscles treated with the anti-sense probe, *Marf mRNA* (magenta) co-localized with NE buds marked by Lamin (Figure 5A, yellow triangle). In contrast, there was low signal and no overlap between the sense *Marf* probe and NE buds in *lacZ* muscles (Figure 5A). These data demonstrate the specificity of the anti-sense *Marf* probe.

We next quantitated foci labeled with *Marf* probes in the myoplasm of control or *pav RNAi* muscles and observed a ~30% reduction in anti-sense *Marf mRNA* in comparison to *lacZ* muscles (Figure 5B and Figure S4E). Minimal signal was seen in *lacZ* muscles treated with sense *Marf* (Figure 5B and Figure S4E), further confirming the probe’s specificity. qPCR was also used to determine *Marf* transcript levels in *pav RNAi* muscles. Consistent with a decrease in *Marf mRNA* foci, there was ~40% decrease in *Marf mRNA* levels upon induction of *pav RNAi* compared to controls (Figure 5C). Note that knockdown of *Marf* transcript using this same method was stronger in *Marf RNAi* compared to *pav RNAi* (Figure S4F). Maybe the presence of an occasional NE budding event in *pav RNAi* muscles allows for the detection of *Marf mRNA* or maybe the cell has an additional and/or alternate route for the nuclear export of *Marf*. However, overall our data argue that loss of Pav reduces the presence of *Marf* transcript in muscles, resulting in mitochondrial abnormalities.

## Discussion

Using a screening approach to identify genes that influence the abundance of p62-linked cargo, we uncovered Pav. Pav and Tum together comprise the centralspindlin protein complex with well-established roles in spindle formation via MT bundling during cytokinesis [21]. Autophagosomal structures marked by p62 and Atg8a were increased upon knockdown of Pav or Tum in larval muscles using RNAi approaches. Further characterization revealed three unexpected results. First, some of the Ubi(+)/p62(+)/Atg8a(+) puncta formed clusters throughout the myoplasm. This clustering phenotype is not observed in other muscle mutants that exhibit elevated numbers of autophagosomes [18,19]. Second, we did not observe Pav or Tum protein in or near the aggregated p62(+) clusters using available antibodies or GFP-tagged versions. Instead, we observed an enrichment of Pav or Tum protein in the nucleus, seemingly inconsistent with a role for these proteins in the myoplasm. Third, p62(+) clusters co-labeled with weakly stained mitochondria, suggesting that a loss of mitochondrial content and/or activity, is sufficient to recruit autophagosomal machinery (Ubi, p62, and Atg8a) for eventual lysosomal turnover.

Our compiled data integrates these findings and together provides a functional model explaining how the knockdown of Pav results in mitochondrial clusters (Figure 5D). Pav participates in the formation of NE buds and *Marf mRNA* is one such cargo that is present at budding foci being exported from muscle nuclei [14,15]. Insufficient transport of *Marf mRNA* to the site of Marf protein synthesis decreases the ability of mitochondria to undergo fusion and the eventual buildup of damaged organelles. Ablation of *Mitofusin 2*, the human homolog of *Drosophila* Marf, also leads to insufficient mitophagy resulting from an accumulation of defective mitochondria in mammalian hearts [38]. Likewise, in *Drosophila* IFMs, knockdown of Marf during pupal development results in mitochondrial dysfunction and engulfment by lysosomes [37]. Although the trigger for the addition of Ubi to mitochondria is not yet clear, polyUbi recruits p62 and Atg8a for autophagosome formation. p62 has been shown to mediate the clustering of dysfunctional mitochondria in HeLa cells via its PB1 domain [39]. A similar mechanism may be functioning in muscle to polymerize defective mitochondria into dense clusters as a protective mechanism or because the aggregates are too large for lysosomal transfer and/or degradation. There may also be involvement of the Wnt pathway since dFz2C is transported to the nuclei and is a known regulator of autophagy [13,40].

Pav participates in the formation of NE buds in at least two larval tissues - salivary glands and bodywall muscle - to export megaRNPs from nuclei [13-15,31]. Established cargo of these RNPs in larval muscles includes *par6* and *magi*, both of which encode for scaffolding proteins required at the postsynaptic NMJ [13]. It is hypothesized that specific transcripts are transported to and locally translated at the NMJ during expansion of these structures during larval development. Since mitochondria have a broader distribution throughout muscle cells [35], the final destination of *Marf* transcripts developing at foci corresponding to NE buds is not clear. FISH analysis of mRNAs present in megaRNPs reveal that a subset of NMJ-associated or mitochondrial-associated transcripts can be detected. Are select transcripts exported in different populations of megaRNPs that have different cellular targets? In the case of mitochondrial-associated mRNAs (such as *Marf*), is the final destination for each megaRNP random or are they targeted to mitochondria in regions of the cell that require more ATP or increased mitochondrial dynamics?

In addition to the weakly stained mitochondria that are marked with p62, we also observed ring-shaped mitochondria in *pav, tum, wash*, and *Torsin RNAi*, but not *lacZ* muscles (Figure S4A). However, these hollow, circular mitochondria did not overlap with p62. Ring-shaped mitochondria are also present in mouse embryonic fibroblasts (MEFs) and HeLa cells after exposure to the protonophore carbonyl cyanide m-chlorophenyl hydrazine (CCCP) for 10 min [41]. This transition from a tubular to globular shape is independent of fission or fusion and may be an intermediate morphology before mitochondrial collapse. Alternatively, additional transcripts that mediate mitochondrial dynamics may also be transported by mega RNPs and thus alter organelle morphology. Indeed, FISH analysis of additional transcripts also showed the presence of *Drp1* transcripts corresponding to NE foci [14].

This study adds a timely perspective on the molecular consequences of NE budding defects. We are the first to study how blocking NE budding causes mitochondrial dysfunction and recruitment of the autophagosome machinery in larval muscles since prior studies focused only on the consequences of age-dependent mitochondrial defects in the *Drosophila* indirect flight muscles (IFMs) [14,15]. The observation that four proteins required for NE bud formation (Pav, Tum, Wash, Torsin) all result in similar mitochondrial morphology defects strongly argues that the transport of mitochondrial-associated mRNAs is crucial for continued mitochondrial health. Moreover, the autophagosomal clusters are a unique phenotype and can be easily examined through immunostaining and microscopy. We believe that our results may provide a simple and rapid method for identifying new proteins that contribute to defects in NE bud development and/or transport in larval muscles.

## Materials and Methods

### Drosophila *genetics and strains*

All stocks were maintained on standard Bloomington *Drosophila* Stock Center (BDSC) cornmeal media at 25°C. Gal4/UAS crosses were set up at 25°C and embryos were collected for 6 hr periods before shifting to 31°C for maximal Gal4/UAS expression. UAS-RNAi lines were obtained from either the BDSC (Bloomington, IN) or the Vienna *Drosophila* Resource Center (VDRC, Vienna, Austria) as indicated: UAS-*mCherry RNAi* (BL35785); UAS*-pav RNAi #1* (v46137); UAS*-pav RNAi #2* (v110330); UAS*-tum RNAi #1* (BL6439); UAS*-tum RNAi #2* (BL67923); UAS*-wash RNAi* (BL62866); UAS*-Torsin RNAi* (BL50620); UAS*-Marf RNAi* (v40478); UAS*-ctp RNAi* (BL44044); UAS*-Dhc64C RNAi* (BL36698); UAS*-Dlc90F RNAi* (BL65189); UAS*-Lis1 RNAi* (BL35043); UAS*-Khc RNAi* (v44338); UAS*-chb RNAi* (BL34669); UAS*-cnn RNAi* (BL35761); UAS*-feo RNAi* (BL28926); UAS*-mud RNAi* (BL35044); UAS*-14-3-3ζ RNAi* (v48724); UAS*-jar RNAi* (BL28064). Additional fly stocks obtained from the BDSC include *Mef2-*Gal4 (BL27390), *da-*Gal4 (BL37291), UAS*-lacZ* (BL3956), *Ubi-p63E-GFP-pav* (BL81651), *sqh-sfGFP-tum* (BL76264), UAS*-GFP-Atg8a* (BL51656), UAS*-GFP-mCh-Atg8a* (BL37749), and UAS-*DsRed-Mito* (BL93056). The *w[1118]* and *y,w* controls are maintained in our lab.

### Western blot analysis

Filleted muscle tissue samples (*Mef2>lacZ* or *Mef2>pav RNAi #1*) were homogenized in SDS sample buffer, followed by heating at 95°C for 3 minutes. The samples were then homogenized, reheated at 95°C for an additional 10 minutes, and centrifuged at 20,000 × g for 1 minute to remove any insoluble debris. The resulting protein lysates were separated via SDS-PAGE and transferred to a nitrocellulose membrane (0.45 µm pore size, Cytiva, Marlborough, MA) using the Trans-Blot® Turbo™ Transfer System (Bio-Rad, Hercules, CA). Membranes were incubated with the primary antibody, rabbit anti-Ref(2)p/p62 (ab178440, 1:2000, Abcam, Cambridge, UK), followed by detection with IRDye 800CW secondary antibodies (1:10,000, LI-COR Biosciences, Lincoln, NE). Revert 700 Total Protein Stain (LI-COR Biosciences, Lincoln, NE) was used as a loading control. Membranes were developed using the LI-COR Odyssey XF system, and protein levels were quantified (Figure 3D) using Empiria Studio Software (LI-COR Biosciences, Lincoln, NE).

### Muscle fillets and immunostaining

Muscle carcass dissections were performed as described previously ([19]). Tissues were stained with the following primary antibodies: rabbit anti-p62/ref(2)p (1:200, ab178440, Abcam, Cambridge, MA), mouse anti-Ubi UBCJ2 (1:1000, ENZ-ABS840, Enzo Life Sciences, Farmingdale, NY), mouse anti-GFP (1:25, 12E6, Developmental Studies Hybridoma Bank, Iowa City, IA), mouse anti-Lamin B (1:200, ADL67.10, Developmental Studies Hybridoma Bank, Iowa City, IA), mouse anti-ATP5α/blw (1:200, ab14748, Abcam, Cambridge, MA), mouse anti-α-tubulin (1:1000, T5168, Sigma-Aldrich, St. Louis, MO), and guinea pig anti-dFz2C (1:2500) [32]. Fluorescence was detected using the following secondary antibodies: Alexa Fluor anti-rabbit, anti-mouse or anti-guinea pig 488 or 594 (1:400, Invitrogen, Waltham, MA). Images were captured using a Zeiss 700 confocal microscope. Image processing and analysis were performed using a combination of Zen Black (Zeiss), ImageJ (NIH), and Adobe Photoshop.

### TMRM and MitoTracker staining

Wandering L3 larvae were dissected live in HL3.1 buffer (70 mm NaCl, 5 mm KCl, 10 mm NaHCO_3_, 5 mm MgCl_2_, 5 mm trehalose, 115 mm sucrose, 5 mm Hepes, pH =7.2). The muscle carcasses were incubated in 100 nM Tetramethylrhodamine ethyl ester perchlorate (TMRM) (HY-D0985A, MCE, Monmouth Junction, NJ) and 1 µM MitoTracker Green FM (M7514, Invitrogen) diluted in HL3.1 for 5 min at room temperature (kept inside a drawer to avoid light) and imaged immediately. Confocal microscopy parameters were kept the same while images were acquired for different samples.

### Magic Red Cathepsin B assay

For the Magic Red Cathepsin B (ImmunoChemistry, Davis, CA) staining, wandering L3 larvae were live dissected in HL3.1 buffer and the internal organs were excised. The remaining muscle carcasses were incubated in a solution of Magic Red (1:250 dilution in HL3.1 buffer) for 20 minutes at 37°C while protected from light by wrapping in aluminum foil. Following a brief wash in PBS, the samples were immediately imaged.

### Fluorescence In Situ Hybridization (FISH) probe synthesis

Digoxigenin (DIG) labeling sense and anti-sense RNA probes was an adaptation from Wilk et al. 2010 [42]. Briefly, 5 µg Marf plasmid (RE04414, Stock 9809, *Drosophila* Genomics Resource Center, Bloomington, IN) was linearized by treating with KasI restriction enzyme (R0544S, NEB, Ipswich, MA) in a 37^°^C water bath for 1 hr. A 1% DNA agarose gel was used to check the digestion quality. The linearized DNA was recovered using the QIAprep Spin Miniprep Kit (#27106, QIAGEN, Hilden, Germany) and the concentration measured using a NanoDrop Microvolume Spectrophotometer (Thermo Fisher Scientific, Waltham, MA). For DIG Labeling of the RNA probe, the following were added to an RNase-free 1.5 mL microcentrifuge tube on ice: 1µg linearized DNA with 2 µl 10x DIG-RNA-Labeling-Mix (11277073910, MilliporeSigma, Burlington, MA), 2 µl 10x RNAPol Reaction Buffer (B9012, NEB, Ipswich, MA), 1 µl SUPERase·In™ RNase Inhibitor (AM2694, Invitrogen, Waltham, MA), 2 µl T7 RNA Polymerase (for sense RNA probe synthesis) (M0251S, NEB, Ipswich, MA) or 2 µl T3 RNA Polymerase (for anti-sense RNA probe synthesis) (M0378S, NEB, Ipswich, MA), and RNase-free water to a final volume of 20 µl. The contents were vortexed, spun briefly, and incubated at 37^°^C for 3 hrs. The volume was then brought to 50 µl using RNase-free water and precipitated by adding 1 µg RNase-free glycogen (R0551, Invitrogen, Waltham, MA), 0.1 vol of 3 M sodium acetate, pH 5.2, and 2.5 vol of cold 100% ethanol. Each sample was vortexed and kept at -80^°^C overnight. The following day the microcentrifuge tube was spun for 20 min at 4^°^C at 12,000 x g and the supernatant was discarded. The pellet was washed with 500 µl cold 70% ethanol and microcentrifuged for 10 minutes at 4^°^C at 12,000 x g. The pellet was allowed to air dry and resuspended in 50 µl RNase-free water. The samples were run on a 1% agarose gel to verify integrity. The probes were stored at -80^°^C until needed.

### *Marf mRNA* detection

We used an established protocol for FISH with minor adaptations [42]. Wandering L3 larvae were live dissected in HL3.1 buffer and fixed in 1x phosphate-buffered saline + 0.1% Tween-20 plus 4% formaldehyde for 20 min. We used Fast Red TR/Naphthol AS-MX(F4648, MilliporeSigma) for the detection of hybridization signals. To do so, we dissolved the Trizma® tablet in 1 mL of distilled water, then added the Fast Red TR/Naphthol AS-MX tablet and vortexed until dissolved. The solution was added to the sample at room temperature for 30 min and kept in the dark to develop the fluorescent signal.

### Quantitative RT-PCR

qPCR samples were prepared as follows. The ubiquitous *da-Gal4* driver was used to knockdown gene expression in all tissues. *yw* was the control. Total RNA was harvested from three whole wandering L3 larvae reared at 31°C. RNA was purified using QuantaBio Extracta Plus RNA kit (Quanta Biosciences, Beverly, MA). Three RNA samples for each genotype were prepared. cDNA was synthesized from 600ng or 1000ng of RNA using the qScript XLT cDNA SuperMix kit (Quanta Biosciences, Beverly, MA). For the qPCR reactions, 1:10 or 1:15 dilutions of the cDNA were combined with PowerUp SYBR Green Master Mix (ThermoFisher, Waltham, MA) and the appropriate primers. qPCR was performed with a QuantStudio 3 instrument and related software (ThermoFisher, Waltham, MA). All primers were used at a final concentration of 1 µM. Primers used to assess autophagosomes and lysosome biogenesis (Figure S2A):

*Atg1* forward 5′-GTCGGGGAATATGAATACAGCTC, *Atg1* reverse 5′-GCATGTGTTTCTTGCGATGAC; *Atg18a* forward 5′-GTGTTCGTCAACTTCAACCAGA, *Atg18a* reverse 5’-TGTCCAGGGTCGAGTCCAC; *Cp1* forward 5′-TCAACTACACTCTGCACAAGC, *Cp1*reverse 5′-GCCAGTCCACAGATTTGGG; *Mitf* forward 5′-AGTATCGGAGTAGATGTGCC AC, *Mitf* reverse 5′-CGCTGAGATATTGCCTCACTTG; *p62* forward 5′-GCCCTCCCAGA ATTACACCA, *p62* reverse 5′-GTTGGCCGAAGAACCCTCT; and *rp49* forward 5′-GCCCAAGG GTATCGACAACA, *rp49* reverse 5′-GCGCTTGTTCGATCCGTAAC. Primers used to validate RNAi knockdowns (Figure S1D,E): *pav* forward 5’-ATGAAGGCAGTACCCAGGAC; *pav* reverse 5’-CGACA GAACACATTCACTGGA; *tum* forward 5’-TCGATGATCTGCGACGCT; *tum* reverse 5’-CTCGTTTTGTATCCTGGCCG. To determine *Marf mRNA* levels in *pav RNAi* muscles (Figure 5C) or *Marf RNAi* muscles (Figure S3E), the following primers were used: *Marf* forward 5′-AGCCGAACATCT TCATCCTGA; *Marf* reverse 5′-TCCTTCTCGTTGCTCACCTT. Larvae were raised at 18°C for the *Marf RNAi* qPCR since they were lethal at higher temperatures.

### Puncta quantification

The Analyze-Particles function in ImageJ was used to identify and quantify the total number of p62 puncta (Figure 1A and Figure S1B), M*arf* probes (Figure 5B), or Magic Red(+) puncta (Figure S2C) in VL3/VL4 muscles combined. The ROI-Manager function was used to measure the overlap between p62 & Ubi (Figure 2C) or GFP-Atg8a & p62 (Figure 2D) puncta in VL3/VL4 muscles combined. PCC analysis of the GFP-mCh-Atg8a autophagy flux reporter was calculated using the Just Another Colocalization Plugin (JACoP) in ImageJ (Figure 3B and Figure S2D,E).

### Mito quantification

The Plot-Profile function in ImageJ was used to assess colocalization of p62 & ATP5α (Figure 4C) or p62 & ATP5α & DsRed-Mito (Figure S4D). The ROI-Manager-Measure function in ImageJ was used to quantitate TMRM and MitoTracker intensity (Figure S4C).

### NE budding quantification

NE buds were counted in muscles VL3 and VL4 from abdominal segments A2 and A3 of wandering L3 larvae. ≥100 nuclei were counted per genotype. Only foci that stained positive for both Lamin B and dFz2C staining were counted as a NE buds. Since NE buds were not detected in all nuclei [10], the number of NE buds within each genotype was randomly separated into three groups using the random function in Excel. Image acquisition and NE bud number quantification were both performed using double-blind strategies.

### Statistical analysis

All datasets were first tested to see if values conformed to a Gaussian distribution using the Normality test function in Prism. The unpaired student t-test was used for pairwise comparisons between two datasets followed by the Mann-Whitney test (if non-Gaussian). All datasets that compared three or more unmatched groups were analyzed using one-way ANOVA with either the Dunnett’s multiple comparisons test or the nonparametric Kruskal-Wallis test. See Table S1 for a summary of statistical tests, sample sizes and p-values.

## Supplementary Material

Supplementary figures_R1_final.docx

S1_Table_Stats_Summary.docx

## Abbreviations

Atg8a: Autophagy-related protein 8a; ATP5α: ATP-Synthetase α; dFz2: *Drosophila* Wingless receptor dFz2; FISH: Fluorescence in situ hybridization; GFP: green fluorescent protein; L3: third instar larva; mCh: mCherry; megaRNP: mega ribonucleoproteins; MT: microtubule; Marf: Mitochondrial assembly regulatory factor; NMJ: neuromuscular junction; NE: nuclear envelope; Pav: Pavarotti; Tum: Tumbleweed; RNAi: RNA interference; TMRM: tetramethylrhodamine methyl ester; Ubi: ubiquitin; Wash: Wiskott-Aldrich Syndrome protein
