# Supplementary material for: Loss of nuclear envelope bud formation leads to mitophagy initiation in Drosophila muscles
Source: Autophagy Rep. 2025 Mar 4;4(1):2471121. doi: 10.1080/27694127.2025.2471121 (PMC11921965; doi:10.1080/27694127.2025.2471121)
Supplement: Supplementary figures_R1_final.docx [file KAUO_A_2471121_SM4812.docx]

**Supplementary Figures (Guo, et al.)**

**
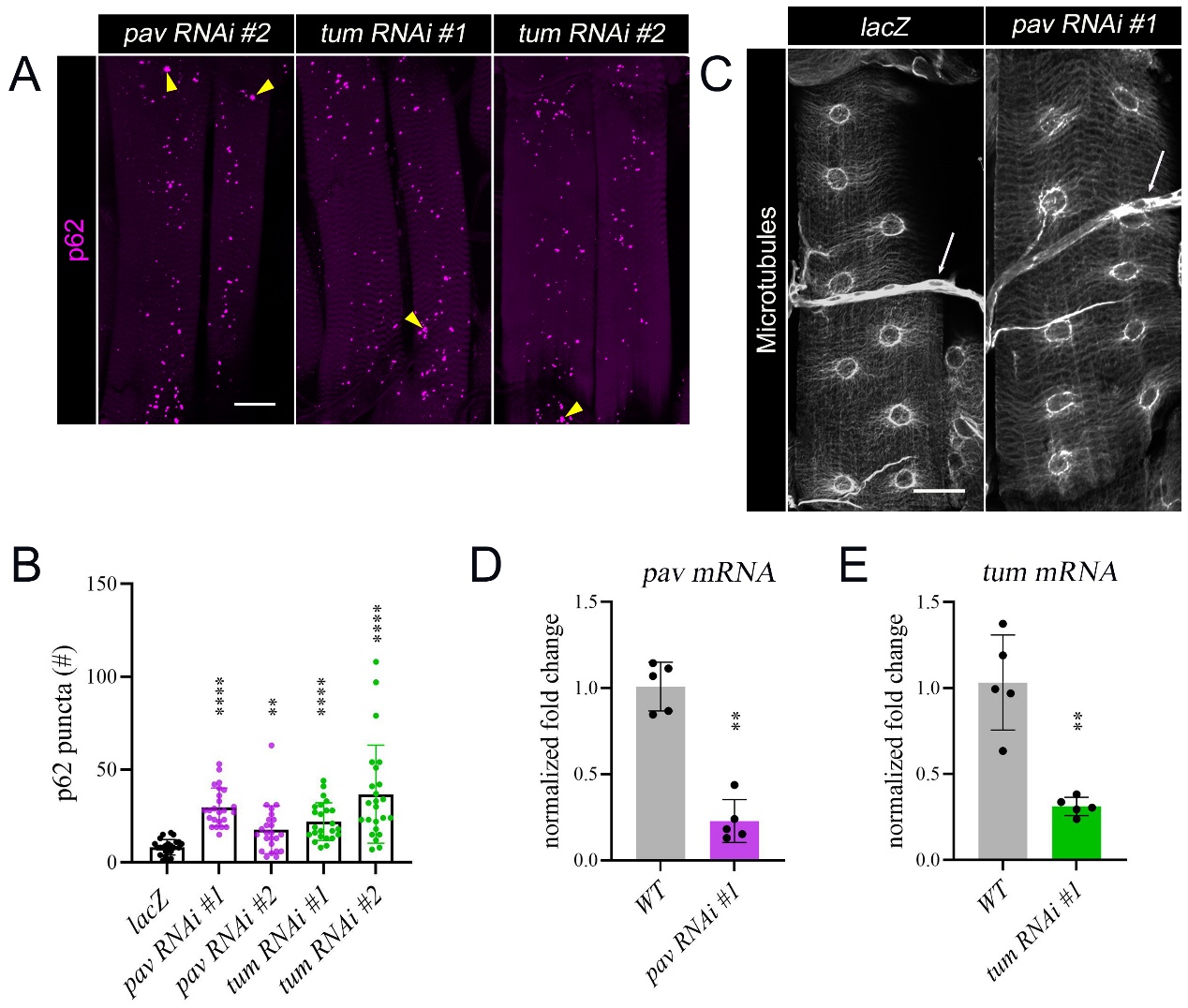
**

**Figure S1.** *tum RNAi* muscles show a clustered p62 phenotype similar to *pav RNAi* muscles. (**A**) Expression of UAS*-pav RNAi #2* or UAS*-tum RNAi #1* and *#2* with the *Mef2-*Gal4 driver. VL3 and VL4 muscles of L3 larvae immunostained with anti-p62 (magenta). p62 clusters (yellow triangle) are observed in *Mef2>pav RNAi #2* and two independent lines expressing *Mef2>tum RNAi*. (**B**) Scatter bar graph shows an elevated number of p62 puncta in the *pav* or *tum RNAi* VL3/VL4 muscles compared to controls. Each data point corresponds to the number of p62 puncta in each VL3/VL4 pair. P-values: **, p<0.05; ****, p<0.001. Error bars indicate standard deviation (SD). N=24. (**C**) Maximum intensity projections of the overall microtubule (gray) pattern in larval somatic body wall muscles. The stained structure indicated by the white arrow is trachea. (**D** and **E**) qRT-PCR was used to quantify the amount of *mRNA* for *pav* or *tum* in whole larvae of control (*y,w*) or *da-Gal4* driven knockdown. *rp49* is used as a normalization control. P values: **, p<0.01; ****, p<0.0001. Error bars indicate standard deviation (SD). N=5 biological replicates. Scale bars, 40 µm (panels A and C).


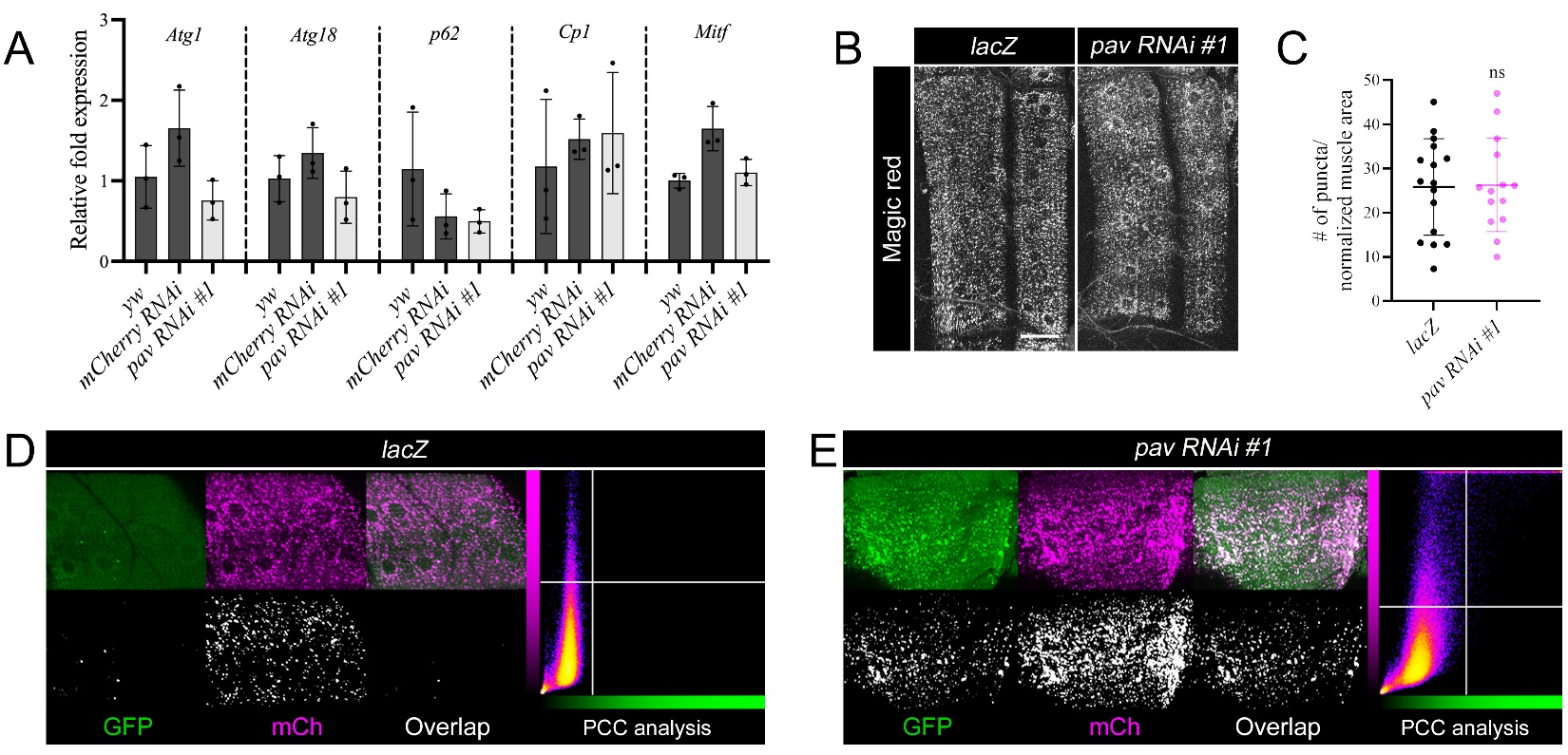


**Figure S2.** Evidence supporting a lack of autophagosome/lysosome biogenesis in Pav knockdown muscles. (**A**) *mRNA* transcripts implicated in autophagosome (*Atg1* or *Atg18*) or lysosome (*Cp1* or *Mitf*,) biogenesis were analyzed using real-time PCR in whole larvae from *y,w* (control), *Mef2>mCherry RNAi* (control), or *Mef2>pav RNAi*. No statistically significant differences in transcript levels are observed for *Atg1*, *Atg18*, *Cp1*, *Mitf*, or *p62*. N= 3 biological replicates. (**B**) Maximum intensity projections of live L3 muscles subjected to a Magic Red assay to measure cathepsin-B as a proxy for lysosome abundance and activity. There is no obvious difference in Magic Red staining between *lacZ* or *pav RNAi* muscles. (**C**) Quantitation of Magic Red fluorescence in panel B shows no difference between control or Pav knockdown muscles. Each data point corresponds to the total number of Magic Red (+) puncta in an area of 1000 µm^2^ selected randomly in VL3 or VL4 muscles. P-value: ns, not significant. Error bars indicate standard deviation (SD). N≥14. (**D** and **E**) Representative images from *lacZ* (B) or *pav RNAi* (B) muscles subjected to Pearson correlation coefficient (PCC) analysis. Maximum intensity stack projections of muscles expressing the GFP-mCh-Atg8a fusion protein (*Mef2>GFP-mCh-Atg8a*). The amount of overlap between the GFP and mCh signals were analyzed using the PCC function in ImageJ. Scale bar, 40 µm (panel B).

**
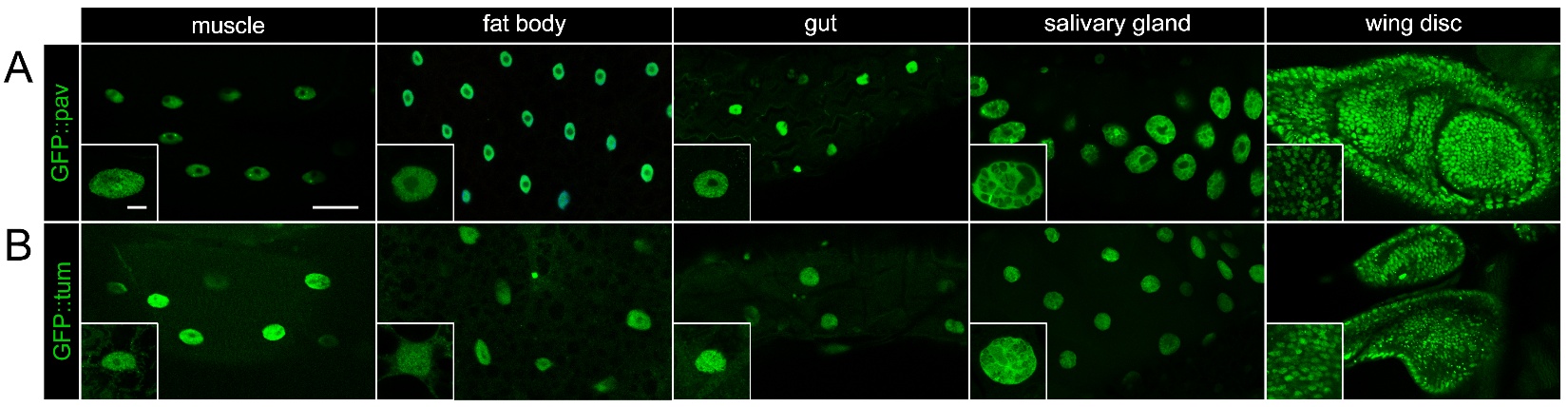
**

**Figure S3.** Pav and Tum expression is predominant in nuclei. (**A** and **B**) Low magnification or high magnification (inset) confocal projections to visualize the location of GFP-tagged Pav (**A**) or GFP-tagged Tum (**B**) proteins. Both proteins are enriched in the nuclei of muscle, fat body, gut, salivary gland, or wing disc tissues. Scale bars, 40 µm or 4 µm in inset.

**
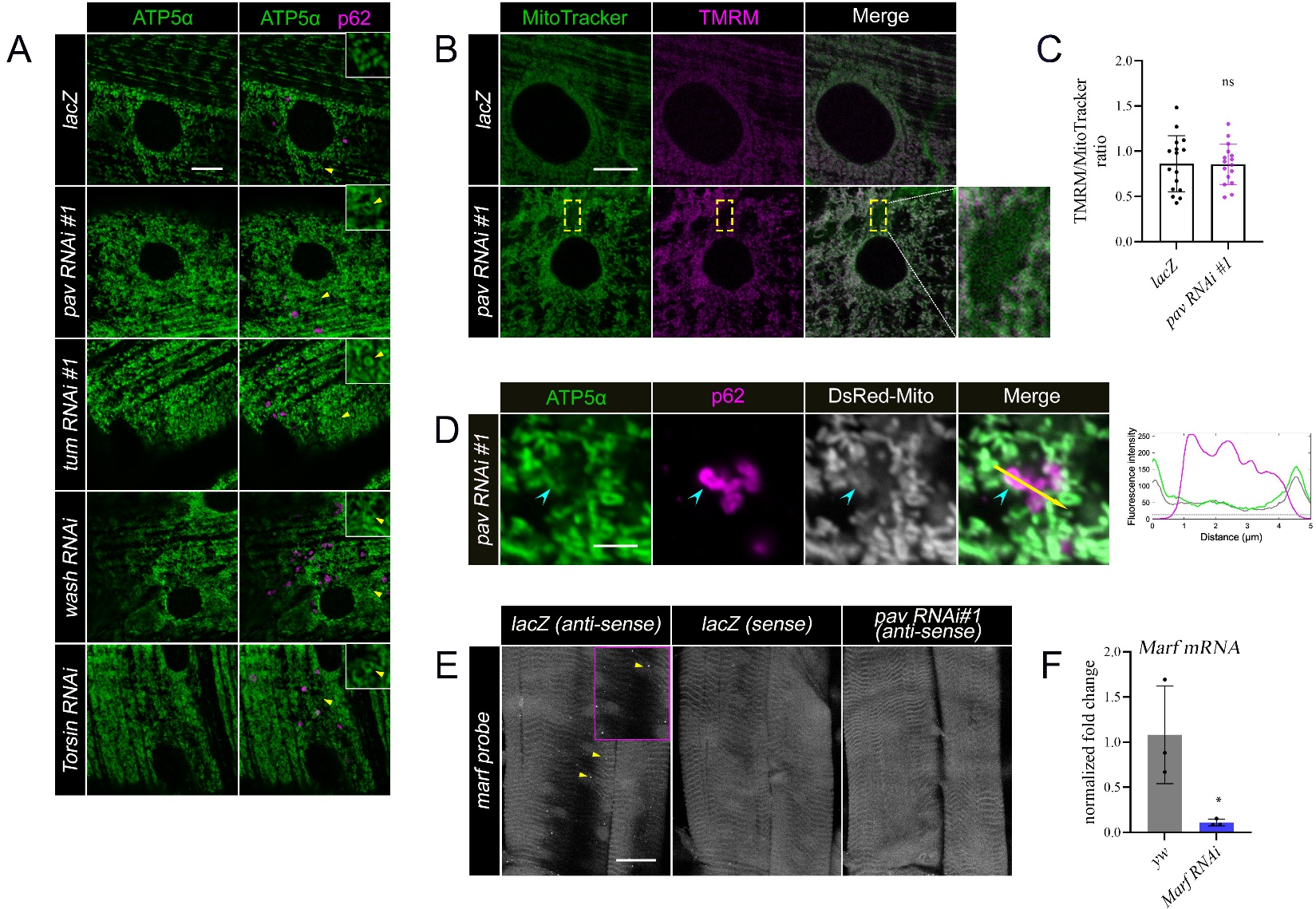
**

**Figure S4.** Mitochondrial integrity, membrane potential, and *marf RNAi* controls. (**A**) Muscles from control or NE budding mutants stained with anti-p62 (magenta) and anti-ATP5α (green). Mitochondria that adopt ring-like structures (yellow triangle and inset) are detected in *pav RNAi #1, tum RNAi #1*, *wash RNAi*, and *Torsin RNAi* muscles but not in control *lacZ* muscles. (**B**) Confocal images of VL3 or VL4 muscles stained with TMRM (indicator of mitochondrial membrane potential) and MitoTracker (indicator of mitochondrial mass). TMRM is detected in mitochondria that also stain positive for Mitotracker. Occasionally, weakly stained mitochondria show decreased membrane potential in *pav RNAi* muscles (inset is a zoom of the yellow rectangular dashed box). (**C**) The overall ratio of TMRM to MitoTracker intensity was calculated in ImageJ from images similar to those in panel B and plotted as a scatter bar graph. No significant difference is detected in *pav RNAi #1* compared with *lacZ* controls. Each data point represents the TMRM/MitoTracker ratio in 400 µm^2^ of L3 muscles. P-value: ns, not significant. Error bars indicate standard deviation (SD). N=16. (**D**) Confocal projections of muscles co-stained with anti-ATP5α (green), anti-p62 (magenta), and DsRed-Mito (gray) in *pav RNAi* muscles. An example of mitochondrial signal that overlaps with p62 is denoted by the cyan indented arrowhead. The corresponding fluorescence plot shows that ATP5α and DsRed-Mito are detectable, but low in areas that co-stain for p62. The gray line corresponds to background levels where no mitochondria are present (see Figure 4C). (**E**) FISH experiments on L3 *lacZ* or *pav RNAi* muscles treated with either anti-sense or sense (control) *Marf* probes as indicated. Puncta corresponding to *Marf* transcripts (yellow triangle), are prevalent in *lacZ* muscles, but reduced in *pav RNAi* muscles. (**F**) qRT-PCR was used to validate *Marf RNAi* knockdown in whole larvae of control (*y,w*) or *da-Gal4* driven knockdown. *rp49* is used as a normalization control. N =3 biological replicates. Larvae were raised at 18^o^C. P value: *, p<0.05. Error bars indicate standard deviation (SD). Scale bars, 10 µm (panels A and B), 3 µm (panel D), 40 µm (panel E).
