# Supplementary material for: Loss of nuclear envelope bud formation leads to mitophagy initiation in Drosophila muscles
Source: Autophagy Rep. 2025 Mar 4;4(1):2471121. doi: 10.1080/27694127.2025.2471121 (PMC11921965; doi:10.1080/27694127.2025.2471121)
Supplement: S1_Table_Stats_Summary.docx [file KAUO_A_2471121_SM4811.docx]

**Table S1. Raw data and statistics summary**

| **Panel** | **Graph type** | **N value** | **Statistical test used** | **Precision** | **p-value(s)^a^** |
| --- | --- | --- | --- | --- | --- |
| Fig. 1A | Scatter plot | N≥15 for each genotype | One-way ANOVA  Kruskal-Wallis test | Median | ns, p<0.01, p<0.005, p<0.001 |
| Fig. 2C | Bar scatter plot | N=12 for each genotype | One-way ANOVA  Kruskal-Wallis test | Mean +/- SD | p<0.005, p<0.001 |
| Fig. 2D | Bar scatter plot | N=24 for each genotype | One-way ANOVA  Kruskal-Wallis test | Mean +/- SD | p<0.001 |
| Fig. 3B | Scatter plot | N≥20 for each genotype | Unpaired student t-test | Mean +/- SD | p<0.001 |
| Fig. 3D | Scatter plot | N=4 for each genotype | Unpaired student t-test | Mean +/- SD | p<0.05 |
| Fig. 4B | Scatter plot | N=3 groups for each genotype | One-way ANOVA  Dunnett’s multiple comparisons test | Mean +/- SD | ns |
| Fig. 4D | Scatter plot | N=3 for each genotype | One-way ANOVA  Dunnett’s multiple comparisons test | Mean +/- SD | ns, p<0.05 |
| Fig. 5B | Bar scatter plot | N=20 for each genotype | One-way ANOVA  Kruskal-Wallis test | Mean +/- SD | p<0.001 |
| Fig. 5C | Column bar graph | N=3 for each genotype | Unpaired Mann-Whitney t-test | Mean +/- SD | p<0.01 |
| **Panel** | **Graph type** | **N value** | **Statistical test used** | **Precision** | **p-value(s)^a^** |
| Fig. S1B | Scatter plot | N=24 for each genotype | One-way ANOVA  Kruskal-Wallis test | Mean +/- SD | P<0.01, p<0.001 |
| Fig. S1D | Column bar graph | N=5 for each genotype | Unpaired Mann-Whitney t-test | Mean +/- SD | p<0.01 |
| Fig. S1E | Column bar graph | N=5 for each genotype | Unpaired Mann-Whitney t-test | Mean +/- SD | p<0.01 |
| Fig. S2A | Column bar graph | N=3 for each genotype | N/A | Mean +/- SD | N/A |
| Fig. S2C | Scatter plot | N≥14 for each genotype | Unpaired Mann-Whitney t-test | Mean +/- SD | ns |
| Fig. S4C | Scatter plot | N=16 | Unpaired Mann-Whitney t-test | Mean +/- SD | ns |
| Fig. S4F | Column bar graph | N=3 for each genotype | Unpaired Mann-Whitney t-test | Mean +/- SD | p<0.05 |

^a^ns = not significant; *, p<0.05; **, p<0.01; ***, p<0.005; ****, p<0.001
